# When diabetes meets a neglected tropical disease: chronic lymphatic filariasis due to *Wuchereria bancrofti*—a case report

**DOI:** 10.1186/s13256-026-06262-x

**Published:** 2026-06-24

**Authors:** V. P. Ansar Ahamed, Abhay Mudey, Abhishek Joshi, T. Athila Jasmine

**Affiliations:** 1 Department of Community Medicine, Jawaharlal Nehru Medical College, Datta Meghe Institute of Higher Education and Research (Deemed to Be University), Sawangi (Meghe), Wardha, Maharashtra 442001 India; 2 Department of Pathology, JSS Academy of Higher Education & Research, (Deemed to Be University), Mysore, Karnataka India

**Keywords:** Lymphatic filariasis, *Wuchereria bancrofti*, Diabetes mellitus, Type 2, Rural health, Case report, India

## Abstract

**Background:**

Lymphatic filariasis (LF) is a neglected tropical disease in some parts of India and results in chronic lymphedema, hydrocele and acute inflammatory episodes. Even outreach to communities often yields undiagnosed cases of chronic cases needing confirmatory testing and tied-in care. This case illustrates the confluence between chronic infectious and metabolic diseases in rural India, and the requirement of comprehensive primary care.

**Case report:**

A 75-year-old South Asian male farmer with type-2 diabetes mellitus detected at a rural camp in the community from rural Kayar, Yavatmal district, Central India, reported to us with chronic swelling of right lower limb and scrotum approximately since 7 years and fever for the past 2 days. We received him in our rural medical college, lab investigations done and blood smear showed microfilariae; ultrasonography confirmed the filarial dance sign, diabetes was associated as a complicating factor. Glycemic optimization, symptomatic treatment for infestation of filariasis and advice on limb hygiene were provided and patient was counseled about vector control and follow-up.

**Conclusion:**

This report highlights the importance of community-based screening camps for detection of chronic LF, confirmation by diagnostic tests at referral centers, and subsequent integration of management of such cases with that for chronic diseases. It highlights the necessity of incorporating neglected tropical disease (NTD) control with noncommunicable disease (NCD) management within rural health programs to strengthen surveillance and sustain progress toward LF elimination targets.

## Introduction

Lymphatic filariasis (LF) commonly known as elephantiasis (Haatipaon), is a serious debilitating disease transmitted through the bite of a Culex mosquito that breeds in dirty/polluted water [1]. LF is a mosquito-borne helminthic infection caused mainly by *Wuchereria bancrofti* (majority of global cases), and less commonly by *Brugia malayi* and *B. timori*. Infection is usually acquired in childhood causing hidden damage to the lymphatic system with visible manifestations (lymphedema, elephantiasis, and scrotal swelling/hydrocele) which occur later in life and can lead to permanent disability [2]. People affected by LF are not only physically disabled, but suffer mental, social and financial losses contributing to stigma and poverty leading to the global burden of neglected tropical diseases [3].

In 2023, 657 million people in 39 countries were living in areas that require preventive chemotherapy to stop the spread of infection [3]. The global baseline estimate of people affected by lymphatic filariasis was 25 million men with hydrocele and over 15 million people with lymphedema. At least 36 million people remain with these chronic disease manifestations. Eliminating lymphatic filariasis can prevent unnecessary suffering and contribute to the reduction of poverty [3].

Lymphatic filariasis (LF), or *Haatipaon*, remains a major public health concern in India and is targeted for elimination by 2027. The disease is endemic in 345 districts across 20 states and union territories, with eight states—Bihar, Chhattisgarh, Jharkhand, Madhya Pradesh, Maharashtra, Odisha, Uttar Pradesh, and West Bengal—accounting for nearly 90% of the national burden. The elimination strategy in India is based on an integrated 5 pronged approach which includes: Mission Mode Mass Drug Administration (MDA), Morbidity Management and Disability Prevention (MMDP) program, vector control measures, multi-stakeholder commitment at the highest level of governance and innovative approaches to resolve programmatic issues [2].

Progress has been significant, with 138 endemic districts passing Transmission Assessment Survey (TAS)-1, while in the remaining 159 endemic districts, MDA is continued annually due to the microfilaria rates greater than 1%. By 2023, 6.19 lakh lymphedema and 1.27 lakh hydrocele cases were reported nationwide. Under the Enhanced Strategy for LF Elimination, MDA campaigns are implemented biannually—on 10 February and 10 August—coinciding with National Deworming Day. In 2023, the two-phase campaign achieved 82% coverage across 170 districts, which rose to 95% in the first phase of 2024 across 96 districts. The August 2024 round covered 63 districts in six states through double- and triple-drug regimens [2].

From a diagnostic standpoint, point-of-care antigen tests such as the filariasis test strip (FTS) for circulating filarial antigen (CFA), microscopy for microfilariae (night blood smears), and ultrasonography demonstrating the “filarial dance sign” are widely used in combination depending on setting and resources. Newer tools—molecular xenomonitoring of mosquitoes (PCR), improved rapid diagnostic test (RDTs), and imaging—help refine surveillance and measure transmission interruption. Therapeutically, mass administration using two-drug regimens (DEC + albendazole) has historically been employed; more recently, WHO has recommended the use of a single-dose triple therapy (ivermectin + diethylcarbamazine + albendazole—“IDA”) in many settings to accelerate elimination, alongside targeted adjunctive regimens such as doxycycline (anti-Wolbachia) for individual patients or clinical trials. Recent research also explores moxidectin combinations and other novel agents [4].

This case highlights the intersection of chronic infectious and metabolic disease in rural India, underscoring the need for integrated primary care. We report this case to highlight community-based detection and integration of LF management with diabetes care. This case report was prepared following the CARE Guidelines [5].

## Case presentation

A 75-year-old South Asian male farmer from Kayar village of Yavatmal district, Maharashtra, India, came with history of chronic right lower limb swelling. He has been an inhabitant of the countryside from a time immemorial and is engaged in the agricultural pursuit to a large extent. He came from a poor family and was nutritionally normal. Past medical history is also remarkable for type 2 diabetes mellitus diagnosed 12 years which was managed on oral hypoglycaemics. The patient’s dwelling nearby cattle sheds and livestock, and stagnant water (a potential site for mosquito breeding) are available near the animal shelters. He lives in a remote house together with his family and reports frequent exposure to the outdoor environment during farming activities.

While attending community health camp at Kayar, the patient presented with complaints of two-day fevers and swelling in the right scrotum along with a long-standing similar swelling over the right lower limb for about seven years. Swelling was gradually progressive and of insidious onset with intermittent febrile spells. There was no previous history of such disease in family or diabetes and he was non addict to tobacco/alcohol. He was evaluated at the camp and after a brief initial examination he was shifted to the rural medical college. He was afebrile and hemodynamically stable on examination at the tertiary center. On local examination, right lower limb showed features of chronic lymphedema with skin thickening and hyperkeratosis. Lymphedema was graded as Stage III (Dreyer classification) [6]. No systemic abnormalities were detected. The right scrotum was enlarged and tender on palpation, consistent with chronic inflammatory changes. There was no evidence of systemic organomegaly, and peripheral pulses were palpable and symmetrical. He had not received previous antifilarial therapy or participated in MDA; diabetes treatment adherence was suboptimal.

General Examination revealed a moderately built, healthy young man with no signs of pallor, icterus, lymphadenopathy, edema, clubbing, dehydration, and cyanosis. His vitals were stable.

**Fig. 1:**
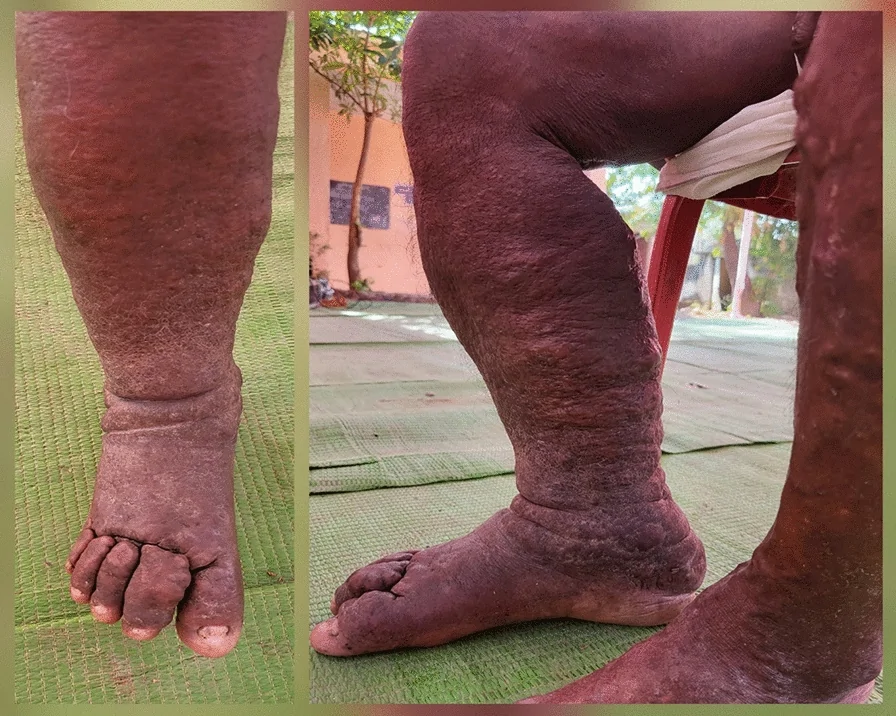
Chronic lymphatic filariasis of the right lower limb showing gross, non-pitting edema extending up to the knee with thickened, hyperpigmented skin, hyperkeratosis and dermal thickening—characteristic of advanced lymphedema

The clinical images (Fig. 1) show the right lower limb of a patient with chronic lymphatic filariasis. The photographs show marked non-pitting edema of the foot to mid-calf, in combination with coarsened and thickened skin and hyperpigmentation characteristic for chronic lymphatic insufficiency. These chronic findings are typical of stage III lymphedema secondary to *Wuchereria bancrofti* infection.

At the rural medical college, routine laboratory investigations were performed. The complete blood count revealed a hemoglobin level of 12.3 g/dL, total leukocyte count of 8600/mm^3^, and a differential count showing neutrophils 62%, lymphocytes 28%, eosinophils 8%, and monocytes 2%, with a platelet count of 2.5 × 10^5^/mm^3^. Mild eosinophilia was noted, consistent with a parasitic etiology. Renal function tests were within normal limits, with blood urea 32 mg/dL, serum creatinine 0.9 mg/dL, serum sodium 138 mEq/L, and serum potassium 4.2 mEq/L. Random blood glucose was 164 mg/dL indicating poor glycemic control in this known diabetic patient. The C-reactive protein (CRP) level was slightly increased at 9.6 mg/L (reference: < 5 mg/L), reflecting a low-grade inflammation in line with a chronic infective process.

**Fig. 2:**
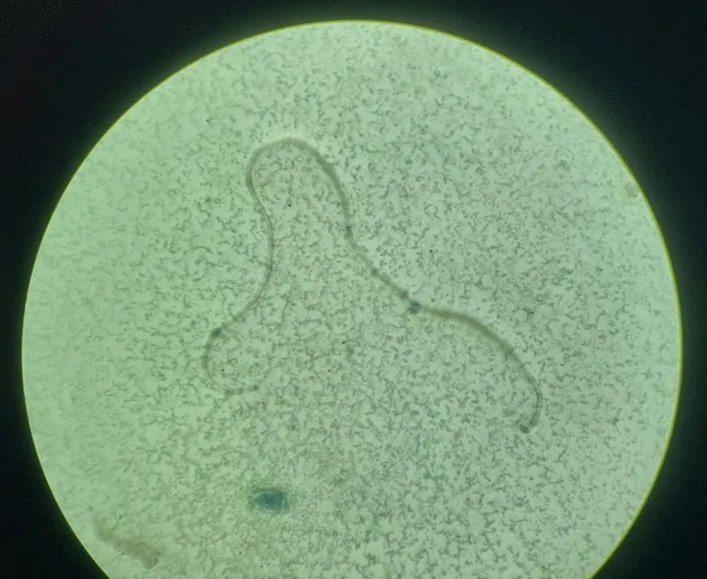
Peripheral blood film (Leishman stain, ×40) revealing microfilaria of Wuchereria bancrofti with an acid-fast sheath and a non-coiled tail, with nuclei not extending to the tip of the tail

Microfilaria was seen in the peripheral blood smear examination after Leishman staining and it resembles with *Wuchereria bancrofti* having a sheath and nuclei not extending to tip of tail (Fig. 2) [7].

**Fig. 3:**
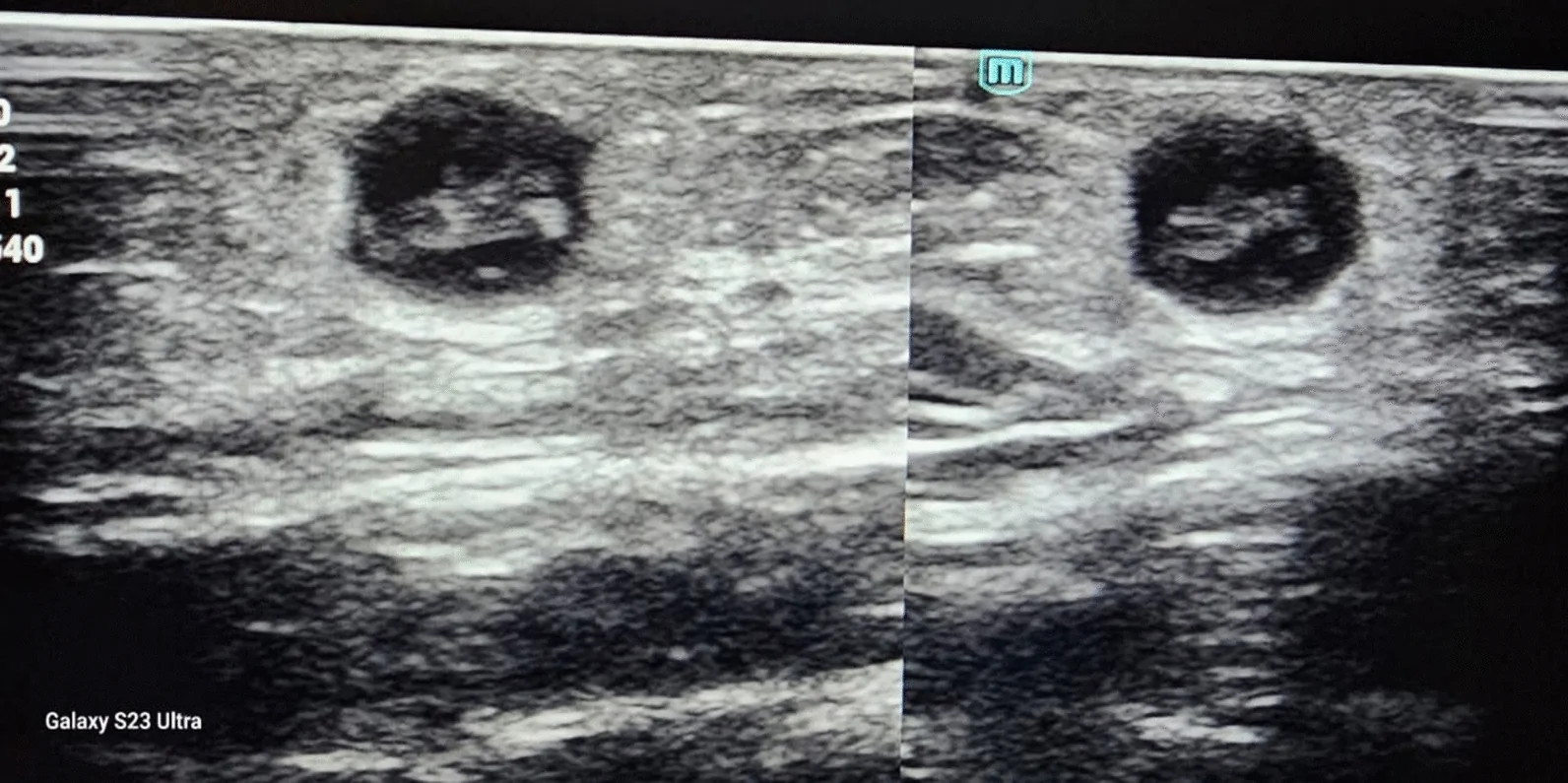
Scrotal ultrasonography demonstrating mobile echogenic structures within dilated lymphatic channels—the characteristic “filarial dance sign” indicating live adult filarial worms in the lymphatic vessels

Sonography of the scrotum revealed movable echogenic structures in enlarged lymphatic channels, also known as ‘filarial dance sign’***.*** This sonographic finding represents the movement of live adult filarial worms within lymphatic vessels and is a well-recognized diagnostic feature in cases of lymphatic filariasis (Fig. 3) [8].

**Final diagnosis:** Chronic lymphatic filariasis involving the right lower limb and scrotum due to *Wuchereria bancrofti*, confirmed by peripheral blood smear and supported by ultrasonographic findings (“filarial dance sign”), in a patient with type 2 diabetes mellitus.

Diagnosis confirmed as per NVBDCP criteria through night blood smear and ultrasonography. Differentials such as diabetic neuropathic edema or DVT were considered but excluded clinically. The combination of microfilariae on smear, filarial dance on USG, and chronic non-pitting edema established the diagnosis.

### Therapeutic intervention

- **Diabetes management:** Review and optimization of oral hypoglycemic therapy, dietary advice, and schedule for follow-up blood glucose monitoring.
- **Filariasis management (individual care and pharmacologic therapy):** Symptomatic care during acute febrile episode—antipyretics, analgesics; advice on limb hygiene, elevation, exercises and skin care to prevent secondary bacterial infection (MMDP principles). Treatment started with Proposed IDA regimen per national guideline: ivermectin 200 µg/kg, DEC 6 mg/kg, albendazole 400 mg single dose. Consideration of antifilarial pharmacotherapy was discussed and planned according to national program guidance and the patient’s overall status/comorbidities [9].
- **Health education:** Counseling regarding mosquito avoidance (nets, environmental management), MDA participation if eligible, and the importance of follow-up for morbidity management.
- **Referral and follow-up plan:** Follow-up planned after one month for blood sugar and lymphatic response. Outpatient follow-up in medicine/community medicine; advice regarding MMDP services and social support where required.

### Follow-up and outcomes

- Patient stabilized and afebrile on discharge from the referral visit. He reported understanding of the diagnosis and agreed to follow-up visits.
- At 1-month follow-up, patient remained afebrile with improved mobility. No side effects during the follow-up initial observation. Antigen test and re-ultrasound planned at 6 months.
- Longer term outcomes (response to antifilarial treatment, progression of lymphedema and compliance with MMDP) will be recorded at follow-up visits. Compliance will be measured through Community Health Worker follow-up.

## Discussion

### Clinical relevance and diagnostic approach

This case demonstrates the typical presentation of chronic LF with chronic unilateral limb lymphedema and scrotal loiasis. Screening in a community camp underscores the importance of outreach services in the endemic/rural areas for case detection and linkage to care. Morphologic identification of microfilariae on peripheral blood smear continues to be useful in some settings for the definitive diagnosis if microscopy is done correctly; however, antigen detection (FTS) and imaging are more commonly used for mapping and case detection. The filarial dance sign on ultrasonography is a specific bedside feature suggestive of live adult worms within the lymph channels and can be used to monitor response to treatment [7].

### Integration with chronic disease care

Integrating it into the management of chronic diseases: if such complications should arise in the elderly patient with diabetes, we have little hope for a good outcome: glycemic control is vital as an emergency measure against secondary staphylococcal cellulitis of lower limbs, so that wound healing and seal immune function are improved. In order to obtain good long-term results, integration of LF morbidity management—this includes skin care, prompt antibiotics for bacterial episodes, elevation and exercise—with chronic disease clinics is crucial in primary health care settings and rural hospital areas. Efforts to eliminate LF are another matter, however. Infection management and disability prevention (MMDP, according to national guidelines) simply cannot be ignored [9].

### Public-health perspective—India and Maharashtra

India has made significant advances toward LF elimination, through a strategy that emphasizes intensified MDA rounds, enhanced coverage and monitoring with rigorous surveillance at the national level. The Government of India’s recent elimination guidelines and enhanced mission-mode MDA (biannual campaigns in many areas) aim for verification of elimination as a public-health problem by the national target year. Nevertheless, residual transmission pockets and chronic cases persist in some districts—necessitating continued case detection, MMDP services and xenomonitoring/surveillance [9].

Specifically for Maharashtra: while several districts have completed MDA and undergone surveillance, some districts historically endemic for LF continue targeted surveillance and MDA efforts where required [9].

### Recent advances in diagnosis

- **Point-of-care antigen tests (FTS):** The Alere/Abbott Filariasis Test Strip (FTS) is a sensitive point-of-care (POC) immunochromatographic assay for circulating filarial antigen; it is widely used for mapping and monitoring MDA impact. Comparative evaluations and newer Rapid Diagnostic Test (RDTs) (for example, STANDARD Q) are being validated to improve field performance. FTS sensitivity and ease of use have transformed program surveillance [7].
- **Ultrasound imaging:** Real-time ultrasound can detect motile adult worms (“filarial dance sign”) in scrotal lymphatics and deep lymphatics and is useful for diagnosis and monitoring of treatment response in selected patients [8].

### Recent advances and evidence in treatment

- **Triple-drug therapy (IDA):** WHO recommends the single-dose triple therapy (ivermectin + DEC + albendazole; IDA) in many endemic settings to accelerate microfilaria clearance and shorten the duration of MDA programs. Implementation is guided by local eligibility (on- elimination mapping, co-endemicity with onchocerciasis, pregnancy, age, and so on). Field evidence shows faster and higher microfilaria clearance with IDA compared to two-drug regimens [10].
- **Anti-Wolbachia therapy (doxycycline):** Doxycycline (4–6 weeks) targets Wolbachia endosymbionts essential for adult worm viability and has macrofilaricidal effects. It is useful as an individual/patient-level option where macrofilaricidal therapy is needed (for example, persistent hydrocele, scrotal worm nests), and as adjunctive therapy in research contexts. Operational limitations (duration, contraindications in pregnancy/children) limit wide MDA use but remain important for clinical care. Recent trials and programmatic studies support doxycycline’s role for morbidity prevention and adult worm sterilization [11].
- **Operational notes:** For an individual patient such as ours, choice of antifilarial therapy should balance comorbidities (for example, diabetes), contraindications (for example, concurrent onchocerciasis), availability of drugs, and program guidance. MMDP and prevention of secondary bacterial infection remain core for limb care independent of anthelmintic therapy [9].

### Limitations and learning points

The case demonstrates the importance of linking community case detection to confirmatory diagnostics at referral centers. As this report describes a single case, the findings cannot be generalized to broader populations. Microfilariae may be intermittent in peripheral blood; hence antigen tests or USG can augment detection [7]. Clear laboratory confirmation and integration with diabetes care is the strength of this case study.

Integration of LF and NCD care is possible, especially in the presence of chronic comorbidity like diabetes, providing for joint treatment with enhanced patient follow-up. Outreach camps support early detection, providing accessible screening and timely referral for neglected conditions like lymphatic filariasis. Adherence to MMDP practices is essential, as consistent limb hygiene and self-care help prevent disease progression and improve long-term outcomes.

## Conclusion

The observation of proven *Wuchereria bancrofti* infection in the rural institution after community detection has been highlighted in this case. It highlights the need for continued community surveillance, effective diagnostic linkage, intensified morbidity management and use of national program guidelines (including the appropriate use of IDA and MMDP) to move toward elimination. Routine district-based surveillance and program updates from NVBDCP/state health authorities will drive public-health action in Maharashtra and India.

## Data Availability

Available from Hospital Records.

## Abbreviations

LF: Lymphatic filariasis
NTD: Neglected tropical disease
NCD: Non-communicable diseases
MDA: Mass drug administration
MMDP: Morbidity management and disability prevention
FTS: Filariasis test strip
CFA: Circulating filarial antigen
PCR: Polymerase chain reaction
DEC: Diethylcarbamazine
IDA: Ivermectin + diethylcarbamazine + albendazole
WHO: World Health Organization
CRP: C-reactive protein
NVBDCP: National vector borne disease control program
DVT: Deep vein thrombosis
RLL: Right lower limb
OHA: Oral hypoglycaemic agents
USG: Ultrasonography
MX: Molecular Xeno monitoring
DNA: Deoxyribonucleic acid
